# An LED-based multi-actinic illumination system for the high throughput study of photosynthetic light responses

**DOI:** 10.7717/peerj.5589

**Published:** 2018-09-04

**Authors:** João Serôdio, William Schmidt, Jörg C. Frommlet, Gregor Christa, Matthew R. Nitschke

**Affiliations:** Department of Biology and CESAM—Centre for Environmental and Marine Studies, University of Aveiro, Aveiro, Portugal

**Keywords:** Chlorophyll fluorescence, Imaging, Photosynthesis, Photoinhibition, Macroalgae, LEDs, 3D printing

## Abstract

The responses of photosynthetic organisms to light stress are of interest for both fundamental and applied research. Functional traits related to the photoinhibition, the light-induced loss of photosynthetic efficiency, are particularly interesting as this process is a key limiting factor of photosynthetic productivity in algae and plants. The quantitative characterization of light responses is often time-consuming and calls for cost-effective high throughput approaches that enable the fast screening of multiple samples. Here we present a novel illumination system based on the concept of ‘multi-actinic imaging’ of *in vivo* chlorophyll fluorescence. The system is based on the combination of an array of individually addressable low power RGBW LEDs and custom-designed well plates, allowing for the independent illumination of 64 samples through the digital manipulation of both exposure duration and light intensity. The illumination system is inexpensive and easily fabricated, based on open source electronics, off-the-shelf components, and 3D-printed parts, and is optimized for imaging of chlorophyll fluorescence. The high-throughput potential of the system is illustrated by assessing the functional diversity in light responses of marine macroalgal species, through the fast and simultaneous determination of kinetic parameters characterizing the response to light stress of multiple samples. Although the presented illumination system was primarily designed for the measurement of phenotypic traits related to photosynthetic activity and photoinhibition, it can be potentially used for a number of alternative applications, including the measurement of chloroplast phototaxis and action spectra, or as the basis for microphotobioreactors.

## Introduction

The description and understanding of how photosynthetic organisms respond to light has long been a central topic in photobiology and photosynthesis research. Light is the primary driver and regulatory factor of photosynthesis, but it is also one of the main stressors of the photosynthetic apparatus. Light causes photoinhibition, the decrease in photosynthetic yield caused by light-induced inactivation of key components of photosystem II (PSII) (Osmond et al., 1997; Tyystjärvi, 2013). The photoinactivation of PSII is in turn counteracted by photoprotective energy-dissipation mechanisms, operating to balance light absorption and damage caused by excess light energy (Müller, Li & Niyogi, 2001; Demmig-Adams & Adams, 2006). The quantitative characterization of the light-dependence of photosynthetic processes is of interest for fundamental research on their underlying mechanisms, their diversity, and the evolution of functional traits (Goss & Lepetit, 2015). However, the interplay between photoprotection and photoinactivation, and of their regulating factors, is also of interest for applied research, as these processes are increasingly recognized as main determinants of plant and algal productivity (Murchie & Niyogi, 2011).

Since its introduction in the 1980’s, Pulse Amplitude Modulated (PAM) fluorometry (Schreiber, Schliwa & Bilger, 1986) has become one of the main techniques used to study photosynthesis and related processes. Based on the active induction of *in vivo* chlorophyll fluorescence through the so-called ‘saturating pulse method’, PAM fluorometry is highly sensitive for photosynthetic activity, and yields parameters closely related to photosynthetic functions. Enabling non-destructive measurements under ambient conditions, this technique has been extensively applied in the study of light stress responses in a wide range of organisms and experimental conditions. Since the introduction of the first PAM fluorometers, designed to be used with leaves or dense microalgal or chloroplast suspensions, new and more sensitive fluorometers have been developed, expanding the use of the technique to the study of dilute suspensions and even single cells, based on optical microscopy (Olson, Chekalyuk & Sosik, 1996; Villareal, 2004) or, more recently, in combination with microfluidics techniques (Erickson & Jimenez, 2013).

An important development was the introduction of imaging fluorometry, which captures images of variable chlorophyll fluorescence induced by saturating pulses (Genty & Meyer, 1994). Originally developed to study spatial heterogeneity in large photosynthetic samples like leaves, lichens, or corals, imaging fluorometry was soon applied to the simultaneous screening of multiple samples (Oxborough, 2004), becoming the basis for the ongoing advances in high-throughput phenotyping of plants and algae (Granier & Vile, 2014; Flood et al., 2016).

More recently, a new method based on chlorophyll fluorescence imaging was introduced which combines the independent illumination of multiple samples and the simultaneous measurement of their photophysiological responses in one single experiment (‘multi-actinic imaging’; Serôdio et al., 2013). By combining the independent control of actinic light intensity and duration of exposure, the method was later extended to the quantitative study of PSII photoinactivation and repair kinetics (Serôdio, Schmidt & Frankenbach, 2017). This method is based on the projection of spatially-separated beams of actinic light on a set of replicated samples, by means of a digital projector. Despite its many advantages, this approach is inherently limited by the complex optical geometry of the projection of actinic light, complicating the relationship between the digitally-set light output levels and the irradiance actually reaching the samples (Serôdio et al., 2013).

Here, we present a novel multi-actinic illumination platform, combining an array of 64 individually addressable LEDs and custom-designed well plates, allowing exposure of samples to photosynthetically relevant light intensities, altogether optimized for imaging of chlorophyll fluorescence. The proposed system is based on open source electronics, off-the-shelf components, and 3D-printed parts, making it inexpensive and simple to fabricate. We demonstrate the application of the system for the high-throughput study of photosynthetic properties of multiple samples by characterizing the response to light stress of several species of marine macroalgae that live sympatrically but have distinct photophysiological traits.

## Materials and Methods

### Design and fabrication

**Figure 1:**
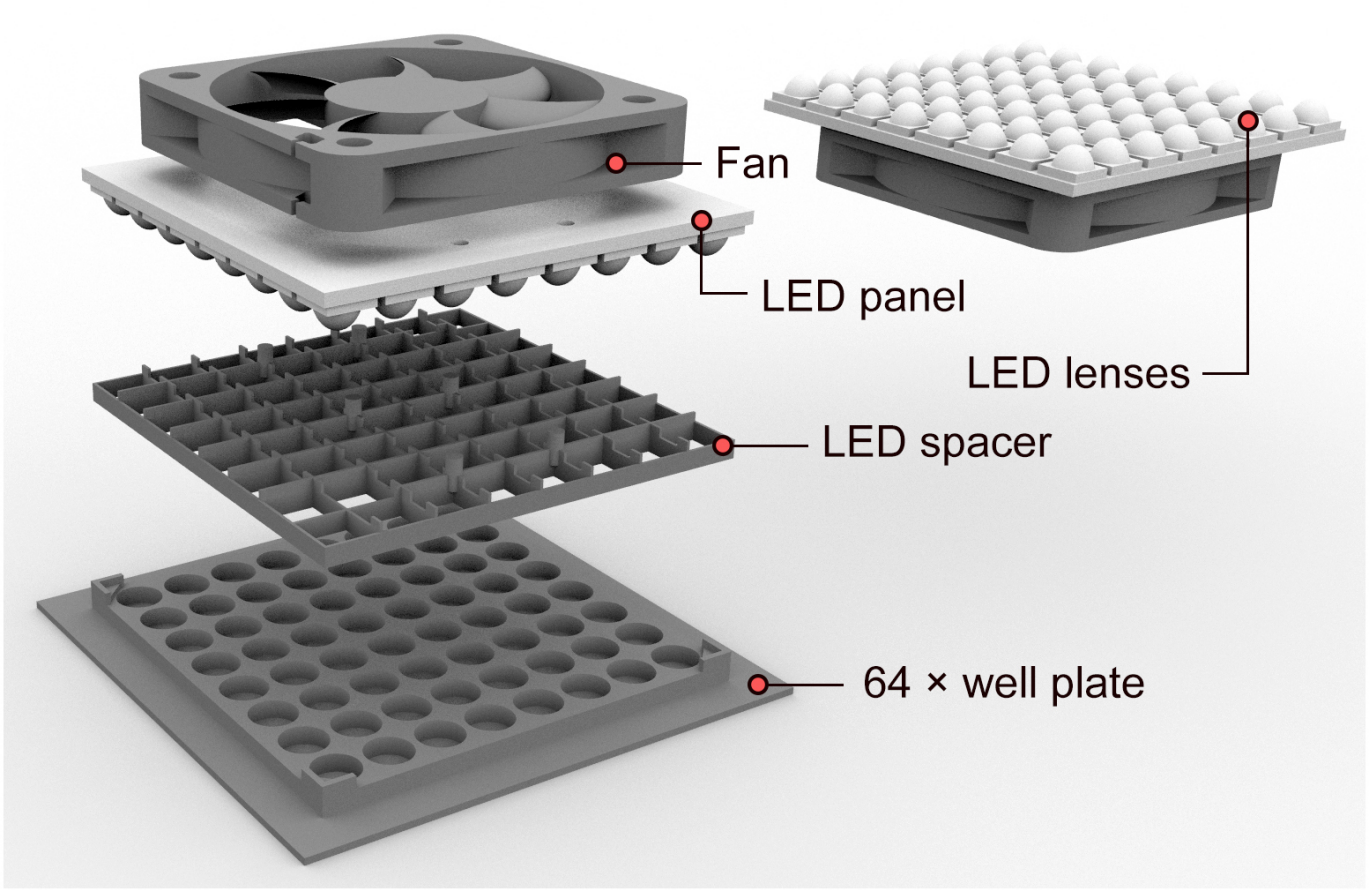
Exploded view of the illumination system, showing main components. Fan to dissipate heat generated by the LED panel; square 8 × 8 SMD LED panel with individual 30° lenses; LED spacer to prevent light spillover between adjacent LEDs; custom-made 64-well plate with guides for centering the LED panel and aligning each LED with its corresponding well. For simplicity, the holders used to fix the fan to the LED panel were omitted, but the corresponding STL files are available as Supplemental Information. The LED panel is placed over the multiwell plate for actinic illumination, being removed during chlorophyll fluorescence measurements.

The illumination system described here is a combination of (i) a set of individually addressable LEDs mounted as an orthogonal array on a flat panel, delivering photosynthetically-relevant intensities of photosynthetically active radiation (PAR), and (ii) custom-made multiwell plates, optimized for independent light exposure of samples and for chlorophyll fluorescence imaging. With access to a 3D printer, the total cost of the system remains below €150. It is comprised of the following components (Fig. 1):

(i) LED panel. A square panel comprising 64 RGBW surface-mounted LEDs (models WS2812B 5050 or SK6812 5050) arranged in an 8 × 8 array (Adafruit NeoPixel NeoMatrix 8 × 8; http://www.adafruit.com). In this study we used the ‘natural white’ LEDs (4500K; http://www.adafruit.com/product/2871) but other models (‘cold’ and ‘warm’ white) are available. The LEDs are individually addressable and were controlled by an Arduino microcontroller (Arduino UNO R3; http://www.arduino.cc), using libraries developed for the Arduino Integrated Development Environment (IDE) (http://www.adafruit.com). The brightness level of each diode can be independently regulated to 256 levels using pulse width modulation (PWM). The LED panel was powered by a dedicated 5V DC 6A power supply (GS60A05-P1J; Mean Well, New Taipei City, Taiwan), as each individual LED draws up to 60 mA at maximum brightness (maximum power consumption of 19.2 W). Despite their low power consumption, these LEDs may be used to deliver photophysiologically relevant intensities, making them adequate for photosynthetic studies. High incident light intensities were achieved by placing the LEDs at a short distance from the samples, while compensating for the close physical proximity with efficient heat dissipation (see below).

(ii) Heat dissipation fan. A 5V DC fan (60 × 60 mm brushless fan; model KD0506PHS2, Sunon, Kaohsiung City, Taiwan) is mounted on the back of the LED panel to dissipate the heat released by the metal plate onto which the LEDs are mounted. The fan is fixed to the LED panel, at a distance of approx. 5 mm, using two 3D-printed parts (File S1) fitted on the mounting holes of the LED panel and of the fan. The use of a 5V fan is advantageous because it can be powered directly from the Arduino microcontroller or from the power adapter used to power the LEDs.

(iii) LED lenses. Each LED is covered with a PMMA transparent (93% transmittance) lens, designed to fit on SMD 5050 LEDs, and to focus emitted light into a 60° beam angle (Shenzhen Glowed Electronic, Shenzhen, China). The lenses increase the irradiance reaching the sample surface by approx. 30%, allowing the positioning of the LEDs at a greater distance from the samples and thus minimizing sample heating.

(iv) LED spacer. A custom-designed LED spacer (File S2) is fitted to the LED panel to prevent light spillover between adjacent LEDs and guarantee the independent illumination of each sample. This part also fixes the position of the LEDs relative to the multiwell plate (see below), ensuring that each LED is correctly positioned above the corresponding well.

(v) Custom-made multiwell plate. Samples are placed in custom-made 64-well plates, with dimensions and well-to-well separation designed to match the spacing between the LEDs (File S3). The multiwell plates have shallow wells (3 mm depth), designed to optimize (i) the exposure of samples to high actinic irradiance levels by allowing a shorter distance between the LEDs and the bottom of the wells, and (ii) the capture of chlorophyll fluorescence images and determination of photophysiological parameters. This design maximizes the sample area illuminated by the fluorometer’s measuring light and saturating pulses (see below), which in some models are projected obliquely (Serôdio et al., 2013). Commercially available 96-well plates (e.g., 96 Well Black Polystyrene Microplate, Corning Incorporated, Tewksbury, MA, USA), although matching closely the spacing of the LEDs of the mentioned LED panels, have wells that may be too deep (>10 mm depth, <7 mm diameter) for chlorophyll imaging, as the shadow cast on the bottom significantly reduces the area usable for measurements. Shallow wells are also advantageous as they allow using lower sample volumes (working volume: 200 µL) and a more economical use of chemicals, like protein synthesis inhibitors. The well plates were 3D-printed using non-fluorescent black polylactic acid (PLA) filament, as described previously (Serôdio, Schmidt & Frankenbach, 2017), minimizing light reflection and scattering. Optionally, the well plates can be fabricated with a mounted water bath jacket, which allows temperature control after connecting to a thermostatic water bath. The well plates are designed so that a 2 mm-gap remains between the surface of the plate and the LED lenses, favoring air circulation and heat dissipation.

(vi) Multi-position holder for Photosynthetically Active Radiation (PAR) sensor (File S4). In order to reliably measure the light reaching the bottom of the various wells of the multiwell plate (important to ascertain LED-to-LED and well-to-well variability regarding illumination conditions) a special multi-position holder was designed to position a mini PAR sensor (see below) directly below the center of each LED, at a distance corresponding to the bottom of the wells. This is also useful for the regular verification of the light output, which can be expected to vary over the lifetime of the LEDs (Glemser et al., 2016).

### PAR irradiance

PAR irradiance was measured using a calibrated flat mini quantum sensor (LS-C and reading unit ULM-500, Heinz Walz, Effeltrich, Germany). Irradiance levels reaching the sample surface were measured using the multi-position PAR sensor holder. PAR irradiance was measured for eight equally spaced LED brightness levels (from 0 to 255). Five randomly selected LEDs were used for each light level.

### LED emission spectra

The emission spectra of the LEDs were measured over the 200–1,025 nm bandwidth, with a spectral resolution of 0.40 nm, using a FLAME spectrometer (FLAME-S-XR1-ES; Ocean Optics, Duiven, The Netherlands) connected to a 400-µm diameter optical fiber with a CC3 cosine corrector made of spectralon diffusing material (QP400-2-UV–VIS-BX, Ocean Optics). The optical fiber was positioned perpendicularly to the LED surface, at a fixed distance set to match the integrated area with the total surface area of the LED. A spectrum measured in the dark was subtracted to the measured spectra to account for the dark current noise of the spectrometer. Spectra were measured for each monochromatic (R, G, B) LED and white (W) separately, and for all four (R, G, B, W) combined. Spectra were also measured for eight equally spaced LED brightness output levels. The full width at half maximum (FWHM) of the peak wavelengths of each LED were calculated from the raw spectral data using the open-source Pavo software (version 1.1.0) (Maia et al., 2013), implemented in the statistical software R (R Core Team, 2003). The *summary.rspec* function was used to determine the relative contributions of spectral ranges (violet, 400–415 nm; blue, 400–510 nm; green, 510–605 nm; yellow, 550–605 nm; and red, 605–700 nm) to the total brightness across the range of intensities applied.

### Temperature

**Figure 2:**
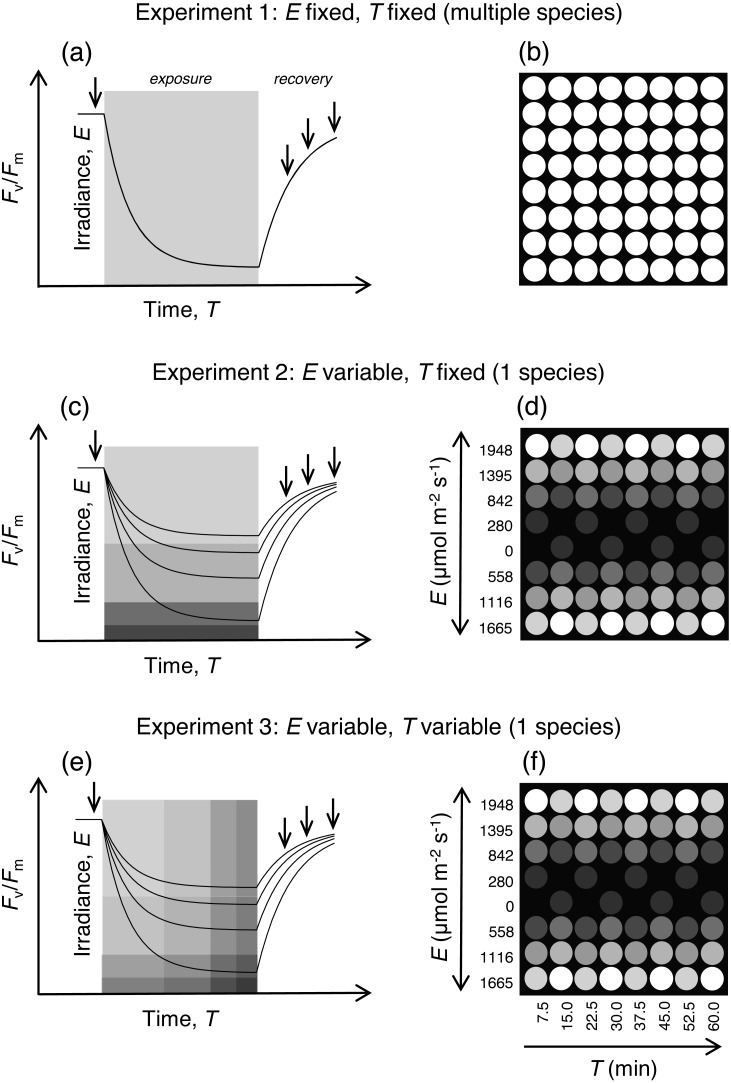
Schematic description of the experiments and corresponding distribution of applied irradiance levels. (A–B) Experiment 1. Irradiance and time of exposure fixed. All LEDs delivering the same irradiance for the same period. (C–D) Experiment 2. Irradiance variable, time fixed. All LEDs turned on at the same time and for the same period, but multiple (eight) irradiance levels are applied. (E–F) Experiment 3. Irradiance variable, time variable. Sets of LEDs comprising eight light levels (corresponding to columns in the figure) are turned on sequentially, with a delay of 7.5 min between consecutive sets, varying both intensity and duration of exposure. (A, C, E) the labels ‘Irradiance’ and ‘Time’ refer to the gray insert where shade of gray indicates light intensity (lighter tone means higher irradiance); lines represent generic responses of *F*_*v*_∕*F*_*m*_; arrows represent measurements of *F*_*v*_∕*F*_*m*_ before and after exposure to actinic light. (B, D, F) the irradiance level is indicated by the different shades of gray the level of each shade of gray is indicated by the correspondence between numbers on the left of the heat maps and the first leftmost column (D, F).

The heating of samples caused by the LEDs was measured by filling the wells with 200 µL of water at room temperature (20 °C) and turning on the LEDs as for carrying out the experiments described below (Fig. 2). The temperature of the water in the wells was measured by a contactless infrared thermometer (factory-calibrated and 0.5 °C accuracy; MLX90614, Melexis, Leper, Belgium), controlled by an Arduino microcontroller (http://www.arduino.cc).

### Imaging of chlorophyll fluorescence

Chlorophyll fluorescence was measured using a FluorCAM 800MF imaging fluorometer (Photon System Instruments, Brno, Czech Republic), comprising a computer operated control unit (SN-FC800-082; Photon System Instruments) and a CCD camera (CCD381; PSI) with a f1.2 (2.8–6 mm) objective (Eneo, Rödermark, Germany). Images of chlorophyll fluorescence parameters *F*_*o*_ (dark-adapted fluorescence level) and *F*_*m*_ (maximum fluorescence) were captured before and after actinic illumination by applying modulated measuring light and saturation pulses (<0.1 and >7,500 µmol photons m^−2^ s^−1^, respectively) provided by red LED panels (612 nm emission peak, 40-nm bandwidth). Images (512 × 512 pixels) were processed and areas of interest (AOIs) were defined by excluding the non-fluorescent background signal using the FluorCam7 software (Photon System Instruments). Fluorescence images were captured at regular time intervals and the values of *F*_*o*_ and *F*_*m*_ were calculated by averaging all pixel values in each AOI (Serôdio, Schmidt & Frankenbach, 2017). The maximum quantum yield of photosystem II (PSII) was calculated as *F*_*v*_∕*F*_m_ = (*F*_m_ − *F*_o_)∕*F*_*m*_ (Schreiber, Schliwa & Bilger, 1986).

### Application

The use of the presented system was illustrated with three experiments, commonly used in photophysiological studies, which apply different combinations of light intensity (*E*) and exposure time (*T*):

(i) Fixed light intensity and fixed period of exposure: multiple parallel light stress-recovery experiments (LSE) (Experiment 1; Fig. 2A). In this type of experimental protocol, one of the most commonly used in photosynthesis studies, *F*_*v*_∕*F*_*m*_ is measured before and after exposure of samples to a pre-defined period of high intensity actinic light, to determine the effects of the applied light stress and to quantify photodamage and recovery capacity (Müller, Li & Niyogi, 2001). Typically, LSEs are conducted on one sample at a time. Here we illustrate the possibility to run multiple LSEs in parallel, allowing for the high-throughput screening of multiple samples. We used samples of various species of marine macroalgae belonging to eight different genera (see below), with the hypothesis that functional diversity exists in their physiological responses to high light. Circular sections of the thalli were cut out using a cork borer (3 mm-diameter). *F*_*v*_∕*F*_*m*_ was measured in the dark, before (for 15 min) and after (for 16 min) exposure to 1,948 µmol photons m^−2^ s^−1^ for 30 min, at 2 min intervals. This period was chosen to allow the relaxation of transient effects on *F*_*v*_∕*F*_*m*_ associated to the xanthophyll cycle, the primary photoprotective mechanisms in eukaryotic photoautotrophs (Christa et al., 2017). Since the LED panel had to be removed to carry out the post-illumination measurements, the first measurement was taken 10 s after the LEDs were turned off. Three replicates of each species were used, each obtained from a different thallus. The kinetics of the *F*_*v*_∕*F*_*m*_ measured during post-illumination recovery, expressed as percentage of pre-illumination levels, were described by fitting a first-order exponential model (Olaizola & Yamamoto, 1994; Serôdio et al., 2012):

(1) \begin{eqnarray*}{F}_{v}/{F}_{m}(t)={F}_{v}/{F}_{m(\max )}+({F}_{v}/{F}_{m(0)}-{F}_{v}/{F}_{m(\max )}){e}^{-{k}_{FvFm}t}\end{eqnarray*}

where *F*_*v*_∕*F*_m(0)_ and *F*_*v*_∕*F*_m(max)_ are the values measured immediately after the end of the light exposure period and the maximum value attained during the recovery period, respectively, and *k*_*FvFm*_ is the rate constant of *F*_*v*_∕*F*_*m*_ recovery. To reduce sample heating, the samples were distributed into the outermost wells placed around the edge of the panel.

(ii) Variable light intensity and fixed period of exposure: light-response of *F*_*v*_∕*F*_*m*_ (Experiment 2; Fig. 2B). The effects of light exposure on *F*_*v*_∕*F*_*m*_ are often used to quantify the intensity of photoinactivation (slowly-reversible damage to PSII) and the counteraction of photoprotective processes (Campbell & Tyystjärvi, 2012). Conventionally, the characterization of the light-dependence of these processes requires the time-consuming exposure of replicated samples to the various light intensities in separate experiments. Here, we demonstrate the possibility of quantifying the response to multiple light levels simultaneously, in a single assay. Eight LSEs (as described above) were carried out in parallel on samples of the green macroalga *Ulva lactuca* L. Three replicates were exposed to each of eight equally-spaced irradiance levels (0 to 1,948 µmol photons m^−2^ s^−1^). To increase heat dissipation and minimize sample heating, the highest light intensities were allocated to the LEDs distributed around the edge of the panel, the LED brightness gradually decreasing towards the center of the panel (Fig. 2B).

(iii) Variable light intensity and variable period of exposure: rate constant of PSII photoinactivation (Experiment 3; Fig. 2C). The presented system can be used to replicate the protocol based on multi-actinic illumination to measure Φ_*PI*_, described in detail previously (*sensu* Serôdio, Schmidt & Frankenbach, 2017). A set of replicated samples are exposed to a range of combinations of light intensity and duration of exposure (‘dynamic light mask’) in such a way that the response to the range of light doses (product of intensity and exposure) can be quantified in detail in a single experiment. A particular use of this type of experiment is the estimation of the rate constant of PSII inactivation (*k*_*PI*_), by measuring the variation of PSII photoinactivation (as measured by *F*_*v*_∕*F*_*m*_, expressed as percentage of pre-illumination levels) with time of exposure (*T*) for a certain irradiance *E*, on samples treated with inhibitors of PSII repair, such as chloramphenicol or lincomycin (Campbell & Tyystjärvi, 2012):

(2) \begin{eqnarray*}{F}_{v}/{F}_{m}(E,T)={F}_{v}/{F}_{m} \left( E,0 \right) {e}^{-{k}_{PI}(E)T}\end{eqnarray*}

The determination of *k*_*PI*_ for various irradiance levels (*E*) allows for an estimate of the quantum yield of PSII photoinactivation (Φ_*PI*_) (Campbell & Tyystjärvi, 2012):

(3) \begin{eqnarray*}{\phi }_{PI}(E)= \frac{{k}_{PI}}{E} \end{eqnarray*}

These inhibitors function by inhibiting chloroplast (prokaryotic) DNA translation and protein synthesis and permit to measure *k*_*PI*_ and Φ_*PI*_ in the absence of PSII repair. Here we demonstrate this approach by using the green macroalga *Bryopsis hypnoides*, exposed to combinations of eight light levels (0 to 951 µmol photons m^−2^ s^−1^) and eight exposure times (applied by the cumulative exposure to periods of 7.5 min, up to a total of 60 min). The samples were treated with chloramphenicol (Sigma-Aldrich, Inc., final concentration 7.7 mM) added in the dark 30 min prior to the start of the experiment.

### Sample collection and cultivation

Specimens of seven species of macroalgae were collected during low tide at an intertidal rocky shore in Praia da Granja, located on the northwest coast of Portugal (41°2′N, 8°39′W) on March 2018. The macroalgae were maintained in aquaria (18 °C, 50 µmol photons m^−2^ s^−1^ provided by fluorescent lamps) with local seawater until the experiments were carried out the next day. The macroalgae species collected were the following: *Bifurcaria bifurcata* R. Ross, *Fucus vesiculosus* L., *Sacchoriza polyschides* (Lightfoot) Batters, and *Sargassum vulgare* C. Agardh (Class Phaeophycea, ‘brown algae’); *Corallina officinalis* L. and *Porphyra leucosticta* Thuret (Rhodophyta, ‘red algae’); *Ulva lactuca* L. (Chlorophyta, ‘green algae’). A second species of Chlorophyta, *Bryopsis hypnoides* J.V.Lamouroux, was obtained and cultured under 200 µmol photons m^−2^ s^−1^ in the laboratory as described elsewhere (Christa et al., 2017).

## Results

### PAR irradiance

**Figure 3:**
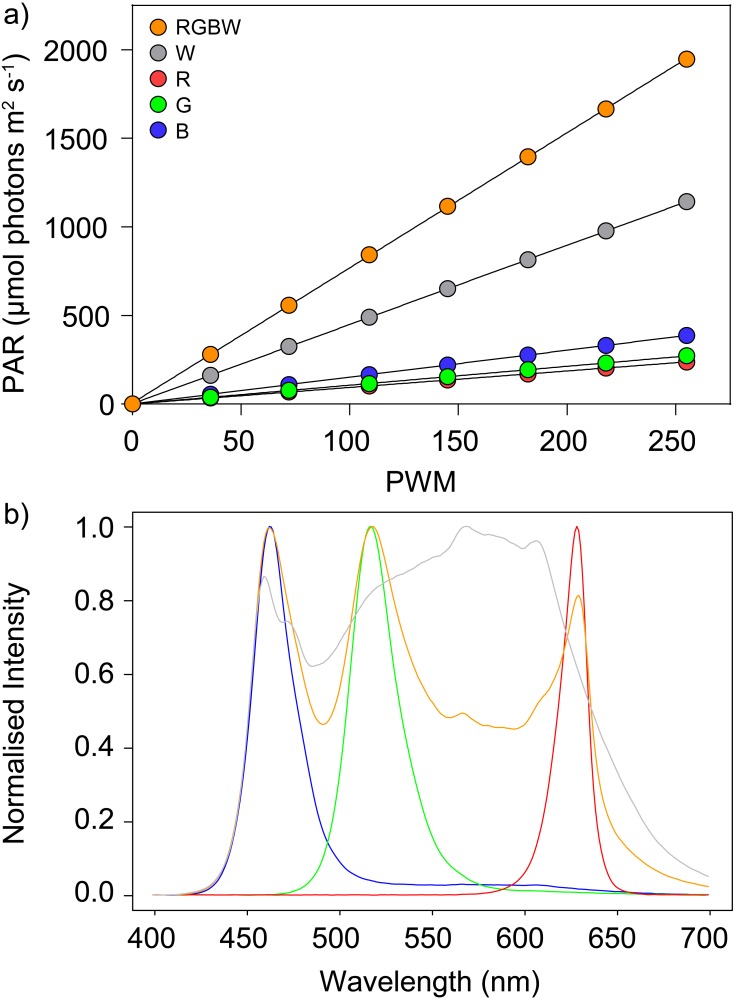
PAR output and spectral composition the LEDs. (A) PAR output of LEDs as a function of PWM level. There is an average of five randomly selected LEDs. Error bars indicate ±1 standard error (hidden behind data points). (B) Spectral output of LEDs. In both cases, measurements for each monochromatic LED (R, G, B) and white (W) separately, and for all four colors (R, G, B, W) combined.

The irradiance emitted by the tested LEDs varied linearly with PWM intensity settings, as defined in the microcontroller code, for all colors, separately and combined (Fig. 3A). Maximum irradiance reaching the samples varied substantially between colors, ranging from 238 µmol photons m^−2^ s^−1^ for red to 388 µmol photons m^−2^ s^−1^ for blue. Using the white LEDs alone resulted in 1,140 µmol photons m^−2^ s^−1^ and the four LEDs combined delivered a maximum of 1,948 µmol photons m^−2^ s^−1^. The tested LED panel showed less than 2% variability in irradiance output between individual LEDs, ensuring low heterogeneity among wells. Also, the output of each LED was verified to be independent from the number and intensity of other LEDs of the panel that were in operation (results not shown). The LED spacer, in combination with the LED lenses, was confirmed to ensure the independent illumination of each individual well by preventing light spillover between adjacent wells. Even when surrounded by wells illuminated by LEDs at their maximum output, the light intensity measured in a well set to be in darkness was <2.0 µmol photons m^−2^ s^−1^.

### Spectra

The output spectra of the red (R), green (G) and blue (B) LEDs were centered at peak wavelengths of 629, 518 and 460 nm, respectively, showing similar values of full width at half maximum (FWHM), ranging from 24 (green) to 27 nm (red) (Fig. 3B; Table S1). The spectrum of the white LED showed a relatively even distribution across the whole PAR wavelength range, covering 425–700 nm, with a broad peak in the yellow region (570 nm), but also with a distinct narrow peak in the blue region (460 nm). The maximum intensity of the white LED (at 570 nm) was significantly lower than the maxima of the R, G and B LEDs, and consequently the combined output of the four RGBW LEDs resulted in the appearance of three distinct peaks corresponding to the R, G and B LEDs. Of importance is that the emitted spectrum of all LEDs was practically independent from output light intensity, as the ratio of blue, green, yellow and red varied <1% across the whole range of output intensities (Table S1).

### Temperature

Operating all LEDs at maximum intensity for extended periods of time (e.g., 60 min) caused an increase in the temperature of the samples. This increase was higher at the center of the plate and reached maximum values around 6 °C. However, this effect was significantly reduced when a gradient of intensities was used, distributing the highest intensities closer to the edge of the well plate (as described in Figs. 2B, 2C). Arranged in this way, even long incubations (60 min) induced a maximum increase of less than 3 °C in central wells, the majority of wells suffering a much smaller increase (<2 °C).

### Experiment 1: light stress-recovery experiments

**Figure 4:**
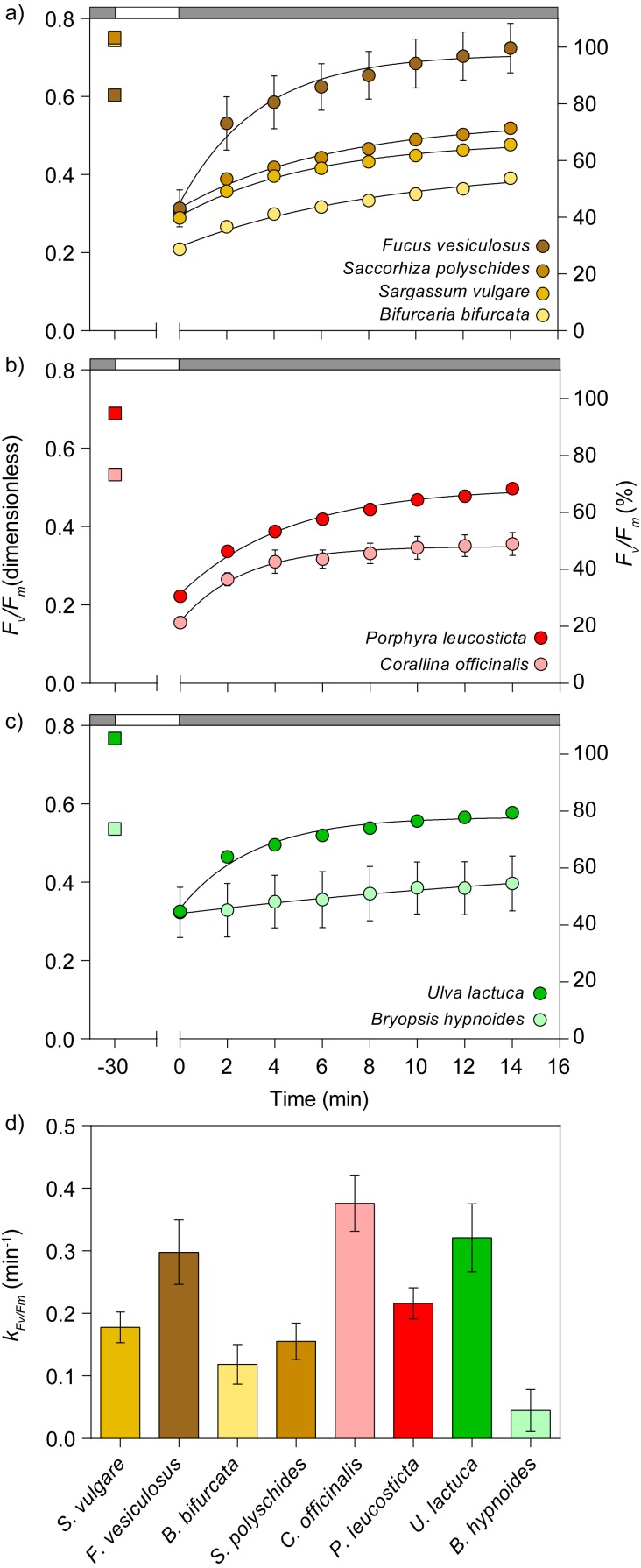
Light stress-recovery experiments carried out simultaneously on eight species of macroalgae (experiment 1). There was a maximum quantum yield of PSII (*F*_*v*_∕*F*_*m*_) of macroalgal samples before (dark-adapted, squares, left axis) and recovery kinetics in the dark for 15 min following exposure to 1,948 µmol photons m^−2^ s^−1^ for 30 min (circles, right axis) (*F*_*v*_∕*F*_*m*_, as percentage of pre-illumination values). Measurements for Phaeophyceae (A), Rodophyta (B), and Chlorophyta algae (C). Lines are the fit of the exponential model of Eq. (1). (D) Rate constants of *F*_*v*_∕*F*_*m*_ recovery (*k*_*FvFm*_) of the studied species. There was an average of three independent measurements. Error bars indicate ±1 standard error (when not shown, hidden behind data points). Gray and white horizontal bars represent darkness and high light exposure, respectively.

The high-throughput potential of the developed illumination system is illustrated by the simultaneous screening of the response to light stress of multiple species of marine macroalgae (Fig. 4). By running multiple LSEs in parallel, it was possible to obtain a detailed characterization of the photophysiology of these species in a single assay. It is worth noting that, if measuring three replicates per species (as was done here), the presented setup has the capacity to test up to 21 different species or sample types. Despite the fact that the tested species originated from the same environment (with exception of *B. hypnoides*), they showed a large diversity in photophysiological states and in their responses to high light stress, as illustrated by the large variation in dark-adapted *F*_*v*_∕*F*_*m*_ values and in *k*_*FvFm*_, respectively. The fit of the first-order exponential model (Eq. (1)) was, in all cases, robust (*r*^2^ ≥ 0.979). *F*_*v*_∕*F*_*m*_ ranged from 0.53 to 0.77. With the exception of *B. hypnoides*, all species recovered following a clear saturation-like pattern, with a fast increase in *F*_*v*_∕*F*_*m*_ in the first 2–4 min, gradually slowing down for the duration of the experiment. *B. hypnoides* is a chlorophyte that was recently found to lack a functional xanthophyll cycle, and showed a low capacity for *F*_*v*_∕*F*_*m*_ recovery following light stress (*k*_*FvFm*_ = 0.05 min^−1^), in clear contrast with the other tested chlorophyte, *U. lactuca*, which exhibited a comparably fast relaxation capacity (*k*_*FvFm*_ = 0.32 min^−1^) (Figs. 4C, 4D).

**Figure 5:**
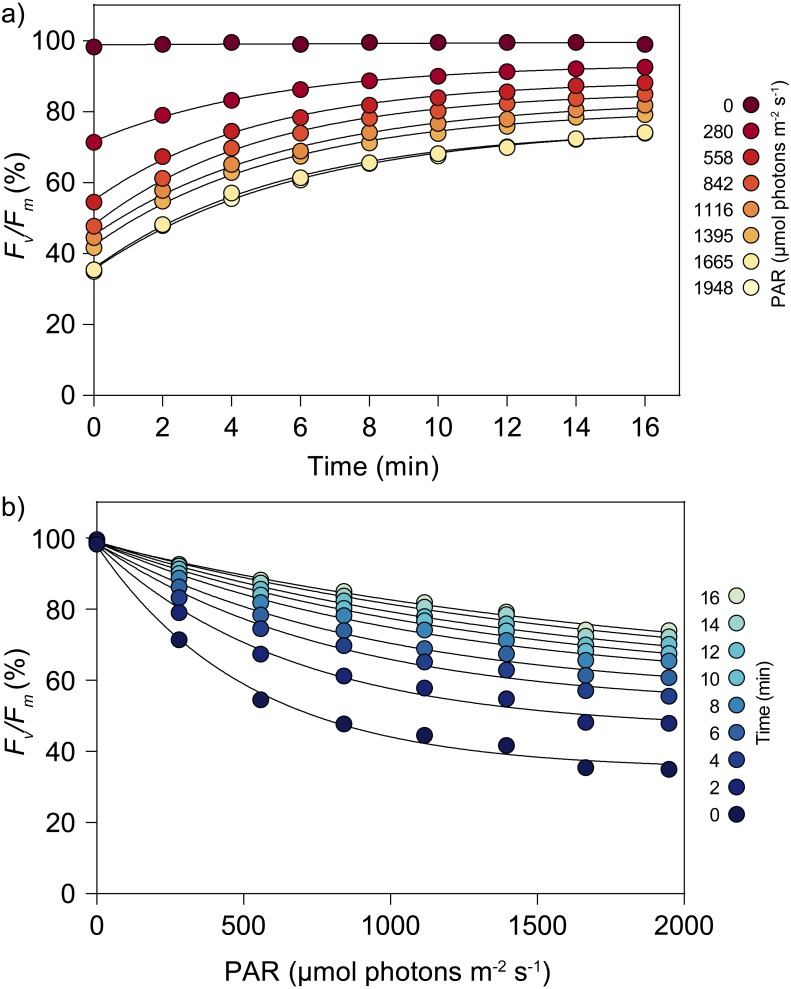
Light stress-recovery experiments carried for eight irradiances on the green alga *Ulva lactuca* (experiment 2). (A) Recovery kinetics following exposure to multiple irradiances for 30 min. (B) Light response of *F*_*v*_∕*F*_*m*_ (%) for different times of recovery. There was an average of three independent measurements. Error bars indicate ±1 standard error (hidden behind data points).

### Experiment 2: light response of *F*_*v*_∕*F*_*m*_ recovery

The possibility of running multiple LSEs in parallel, each one for a different light intensity, allowed for the high-throughput characterization of the light response of *F*_*v*_∕*F*_*m*_ recovery kinetics. Light-response curves of *F*_*v*_∕*F*_*m*_ (% of initial values) could be generated in the time period corresponding to the generation of a single light curve. The results obtained with *U. lactuca* samples illustrated the possibility to quantitatively describe how *F*_*v*_∕*F*_*m*_ varies with time for a range of light intensities (Fig. 5A) and how *F*_*v*_∕*F*_*m*_ varies with light intensity for a time series of post-stress recovery (Fig. 5B). These results extend those observed in the first experiment by detailing the light dependency of *F*_*v*_∕*F*_*m*_ recovery. For this particular species, the capacity for recovery clearly depended on the intensity of the light applied, and the maximum time required for samples to recover >70% of the initial *F*_*v*_∕*F*_*m*_ (across all light-dose treatments) was at least 16 min.

### Experiment 3: rate constant of PSII photoinactivation

**Figure 6:**
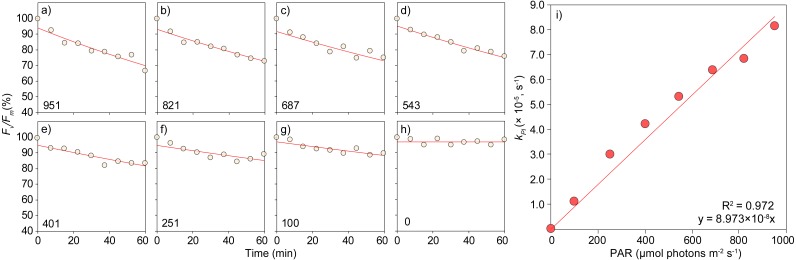
Determination of the rate constant of photoinactivation, *k*_*PI*_, for eight irradiance levels and of the relative quantum yield of PSII photoinactivation, Φ_*PI*_, on the green alga *Bryopsis hypnoides* (experiment 3). (A–H) Variation with time of exposure of *F*_*v*_∕*F*_*m*_ (% of pre-illumination levels) of chloramphenicol-treated samples exposed to multiple irradiances. Lines represent fitted exponential model used to measure *k*_*PI*_ (Eq. (2)). Numbers at bottom-left corner represent the irradiance applied in µmol photons m^−2^ s^−1^. (I) Linear variation of *k*_*PI*_ with irradiance. Line represent linear regression equation, the slope of which is Φ_*PI*_.

The high throughput capability of the illumination system for the characterization of the dependency of PSII photoinactivation on light intensity and time of exposure is illustrated by the simultaneous tracing of *F*_*v*_∕*F*_*m*_ decay over a large range of intensities (Figs. 6A–6H). In all cases, a well-defined time-series of *F*_*v*_∕*F*_*m*_ was measured, allowing for a robust fit of the first-order exponential model for PSII photoinactivation (Eq. (2)), and for an accurate estimation of the rate constant of photoinactivation, *k*_*PI*_, for each light level. The finding of a significant linear correlation of *k*_*PI*_ with irradiance enabled the estimation of the quantum yield of PSII photoinactivation, Φ_*PI*_, given by the slope of the regression equation of *k*_*PI*_ with *E* (Eq. (3), Fig. 6I).

## Discussion

The illumination system described here was designed to be applied in the framework of the multi-actinic imaging approach that was previously established for high throughput photosynthesis studies (Serôdio et al., 2013; Serôdio, Schmidt & Frankenbach, 2017). The central and distinctive features of this approach are: (i) the simultaneous application of programmable light beams of different intensity (or color) and duration to a set of replicated samples and (ii) the subsequent measurement of their photosynthetic response to the applied light treatment using chlorophyll fluorescence imaging. This study shows that the presented LED-based system verifies the main assumptions of the multi-actinic approach related to sample illumination and measurement of important photophysiological responses:

### Range of light intensity and spectra

The system allows for the simultaneous application of a wide range of PAR irradiance levels, from close to complete darkness to values approaching the maximum intensities that can occur under natural conditions (ca. 2,000 µmol photons m^−2^ s^−1^). The possibility to reach PAR intensities of up to 1,948 µmol photons m^−2^ s^−1^ enables the study of responses to levels of light stress that are physiologically realistic, especially considering that photosynthetic processes often saturate under lower irradiances. Furthermore, the possibility to assign adjacent wells as “bright” or “dark” treatments represents an improvement relative to the previously described multi-actinic setup, which used a digital projector as source of actinic light (Serôdio et al., 2013). This was made possible by the design of an LED spacer, which was shown to effectively prevent light spillover between neighboring wells. Another advantage relative to a projector-based system is that the LED panel provides a fixed optical geometry, meaning that the distance between each LED and the corresponding sample, as well as the irradiance angle, is the same for all LEDs. As a result, the calibration of light intensities is improved by a significant margin, based on the establishment of a linear relationship between PWM levels and PAR incidence (as in Fig. 3).

An important prerequisite for the multi-actinic approach is that the spectrum of actinic light does not change with output irradiance. The use of LEDs, and their constant spectrum independent of applied intensity, surpasses a limitation of the original, projector-based system, which required the correction of the spectrum across the range of applied intensities (Serôdio et al., 2013). Furthermore, the inclusion of white LEDs contributes to an overall light spectrum that is desirable for photosynthesis studies, and being relatively rich in yellow and green, is more similar to halogen lamps or sunlight than other systems that use single color, monochromatic LEDs.

### Cold light

The tests carried out showed that, despite the short distance between the LEDs and the wells, the samples experience only a limited increase in temperature, one not expected to have a significant effect on photosynthetic activity, and being acceptable for most photophysiological studies. Temperature changes are known to alter photoinhibition rates mostly through a decrease in PSII repair capacity rather than through direct effects on PSII inactivation (Murata et al., 2007; Campbell & Tyystjärvi, 2012), with low temperatures having a stronger effect than moderate heat (Nishiyama, Allakhverdiev & Murata, 2006). Experimental data have repeatedly shown that PSII photoinactivation is only marginally affected by temperature, even when considering broad variations of more than 20 °C (Tyystjärvi, 2013). Cooling of the samples was achieved through the combined distribution of those LEDs delivering the higher light intensities, and the positioning of the LED panel above the samples, favoring the upward dissipation of hot air, facilitated by the fan at the top. In addition, these effects can be further minimized by using a water jacket connected to a thermostatic bath, or by using a Peltier device (Johnson & Sheldon, 2007). The results of Experiments 2 and 3, showing patterns of light and time-dependent responses identical to the ones commonly observed from independent experiments, carried out at the same temperature, indicate that potential differences in temperature between samples did not affect substantially affect the sample physiology and did not compromise the usefulness of the system. For experiments of type 1 (Fig. 2A), it is recommended that, in the absence of external cooling source, the number and output of LEDs to be used is reduced, and their potential effects on sample heating is ascertained a priori. Heating effects can also be significantly reduced by restricting the LEDs in use (especially if delivering very high intensities) to the positions located at the edge of the panel.

### Chlorophyll fluorescence imaging

The measurement of algal photophysiological responses by imaging of chlorophyll fluorescence is facilitated by the proposed system. The shallow custom-made multiwell plates ensure that the measuring light and the saturating pulses reach the bottom of the wells with minimum shading. This is important in the case of samples like the ones used in this study that settle on the bottom of the wells, and is particularly advantageous for commercially-available fluorometers for which the measuring and saturating light are incident on the samples at an angle (Serôdio et al., 2013). Also in the case of suspensions of microalgae, where cells may tend to accumulate at the bottom of the wells during prolonged incubations, shallow wells are advantageous. Furthermore, the small footprint of the custom-made well plate helps to improve the homogeneity of the light field of measuring light and saturating pulses, and reduces the optical effects of lens distortion, which is typically higher at the edges of the images.

### Applications

As exemplified by Experiment 1 (Fig. 4), the herein presented approach is particularly useful for exploratory studies. It enables the fast screening of multiple samples, the characterization of key photophysiological traits, and the determination of optimal *E* and *T* levels for subsequent experiments. The potential of this technique was demonstrated by the screening of basic physiological states (*F*_*v*_∕*F*_*m*_) and detailed responses to high light stress for several species of macroalgae of different taxonomic groups, growing sympatrically in tidal pools. These include the responses observed immediately following light exposure, which provide insight on the relaxation of photoprotective energy-dissipating mechanisms activated during light exposure (energy-dependent fluorescence quenching), and on the magnitude of the persistent, photoinhibitory damage (photoinhibitory quenching). The former are largely related to the operation of the xanthophyll cycle, which is commonly assumed to be reverted during the first 10–15 min, and the latter is typically ascertained by the difference between *F*_*v*_∕*F*_*m*_ measured at this point and pre-stress levels (Müller, Li & Niyogi, 2001).

The responses to high light (as measured by *k*_*FvFm*_) varied markedly between the tested species, although no systematic difference was found between groups (brown, red, green algae). The large variability observed within brown algae species was equivalent or larger than the overall variability between all species. An obvious outlier was the xanthophyll cycle-deficient species *B. hypnoides*, which could be clearly identified based on its limited capacity for *F*_*v*_∕*F*_*m*_ recovery following illumination (Christa et al., 2017; Giovagnetti et al., 2018). In contrast, *U. lactuca*, also a chlorophyte but one that has a functional xanthophyll cycle, exhibited a comparatively rapid recovery. Such large diversity in the responses to light stress despite the fact that all specimens (exception of *B. hypnoides*) originated from the same intertidal site, may reflect differences in inherent functional photophysiological traits or be the result of phenotypic acclimation to local light and temperature conditions within the small-scale heterogeneous environment of tidal pools (Helmuth et al., 2006).

The response to light stress is strongly affected by the photoacclimation state (Miyata, Noguchi & Terashima, 2012), which may change rapidly as samples are transferred to laboratory conditions. In this context, the possibility to survey a large number of samples simultaneously is advantageous as it ensures that all samples are studied after experiencing the same conditions following collection. Also of importance is the fact that the developed illumination system could be used to investigate even more diverse assemblages (up to 21 species in triplicate) or to rapidly characterize a species across a gradient of natural light environments (e.g., collected from a range of depths) to detect functional differences in acclimation states.

As demonstrated in Experiments 2 and 3, of increasing complexity, the system has the potential for the high throughput characterization of photosynthetic performance despite the lack of measurements during light exposure. LSEs can be expanded to characterize how the samples under study react to both actinic light intensity and duration of exposure, enabling the study of light dose effects. Furthermore, it enables a complete characterization of the light-response of PSII photoinactivation in a single experiment, which represents a more efficient approach than the conventional method of separately measuring individual samples exposed to each combination of intensity and time of exposure. The quantum yield of PSII photoinactivation measured in this study (Φ_*PI*_ = 8.97 × 10^−8^ m^2^ µmol^−1^) is, to our knowledge, the first estimate produced for macroalgae, being well within the range of variation observed for plant leaves (Lee, Hong & Chow, 2001; Sarvikas et al., 2006).

In this study, the irradiance dependency of *k*_*PI*_ was investigated by exposing replicated samples to different irradiances. While this is the most commonly used protocol for studying the *k*_*PI*_ vs *E* relationship and determining Φ_*PI*_ (Tyystjärvi & Aro, 1996; Campbell & Tyystjärvi, 2012), this relationship has also been investigated using samples grown under a range of different irradiances (Baroli & Melis, 1996). The presented system can be readily adapted to this type of approach by regulating the output of the LEDs in order to match the various growth irradiances.

### Limitations

Because the LED panel must cover the samples during illumination, it is not possible to carry out fluorescence measurements simultaneously with actinic light exposure. In practice, this excludes the possibility of measuring light-response curves of fluorescence parameters of other commonly measured indices (Serôdio et al., 2013). However, the separation of exposure to actinic light and the measurement of the fluorescence response does not prevent the carrying out of light stress-recovery experiments, which are informative of key features of the photosynthetic light response. LSEs are the basis of the long-established experimental framework for characterizing the fast (photoprotective) and slow (photoinhibitory) aspects of post-illumination recovery of *F*_*v*_∕*F*_*m*_ (Horton & Hague, 1988; Walters et al., 1991; Müller, Li & Niyogi, 2001). LSEs are, however, typically based on a single combination of one actinic light intensity (*E*) and one exposure period (*T*), both arbitrarily defined *a priori* to induce significant stress. The multi-actinic approach allows for the expansion of LSEs by simultaneously applying multiple *E* × *T* combinations, thus covering a wide range of physiologically-relevant intensities and exposure periods.

A more general limitation of this system is the fact that all photophysiological measurements are based on incident, and not absorbed, irradiance. By not considering the fraction of incident light that is actually absorbed by the samples, this may potentially cause significant deviations in the estimation of parameters like *k*_*PI*_ or Φ_*PI*_ in the cases of optically dense samples or compromise the comparison between samples with very different spectral absorption properties due to dissimilar pigment compositions (Schreiber & Klughammer, 2013). This issue is, however, not specific to the described image-based system, but is an inherent problem when studying optically dense samples like leaves or corals (Campbell & Tyystjärvi, 2012; Serôdio, Schmidt & Frankenbach, 2017; Nitschke et al., 2018).

### Comparison with other multi-sample illumination systems

A number of systems have been proposed, which, combining the use of multiple samples and independent illumination, could potentially be used for multi-actinic fluorescence measurements (Table 1). With the exception of the ‘multiplexed pixel-based irradiance platform’ (Graham, Riordon & Sinton, 2015), all systems are based on LEDs, ensuring the independent illumination of each sample. However, in the case of the ‘PhotoBiobox’ (Heo et al., 2015), the low replication of LEDs (considering the total number of samples) and the apparent lack of individual intensity regulation makes it inadequate for multi-actinic purposes. Most systems were designed and optimized for algal growth (Chen et al., 2012; Graham, Riordon & Sinton, 2015; Heo et al., 2015; Morschett et al., 2017), therefore delivering relatively low operating light intensities. Only the ‘microphotosynthetron’ (Johnson & Sheldon, 2007) was originally designed for measuring photosynthetic light responses, but the maximum light intensity (250 µmol photons m^−2^ s^−1^) is considerably lower than the maximum PAR levels observed in natural conditions and lower than the levels attainable with the system proposed in this study. The various systems differ in several aspects, but most rely on the use of standard 96-well plates (or higher number of smaller wells (Graham, Riordon & Sinton, 2015)) which, as explained above, may cause difficulties in the measurement of chlorophyll fluorescence due to the depth of the wells and the consequent shadowing of excitation light.

**Table 1:** Comparison of features of illumination systems for multiwell plates.

System	Micro-photosynthetron (Johnson & Sheldon, 2007)	Optical microplate (Chen et al., 2012)	Multiplexed pixel-based irradiance platform (Graham, Riordon & Sinton, 2015)	PhotoBiobox (Heo et al., 2015)	Light Plate Apparatus (Gerhardt et al., 2016)	Micro-photobioreactor (Morschett et al., 2017)	This study
Maximum intensity (operating intensity)^a^	250	^e^	148	650	1,057	620 (200)	1,948
LEDs per sample	1	1	n.a.^f^	0.75	2	1	4
LED colors^b^	470	650	n.a.^f^	‘white’	405–699^h^	450, Near-UV, ‘warm white’	460, 518, 629, ‘white’
Intensity levels	12	128	16	Continuous	4,096	??	256
Cost/Engineer expertise	High/Yes	High/Yes	High/Yes	Low/Yes	Low/Yes	High/Yes	Low/No
Number of samples	96	96	238, 1,260	96	24	48	64
Usable for chlorophyll fluorescence imaging	Limited^d^	Limited^d^	No^g^	Limited^d^	Yes	No^d^	Yes
Heat dissipation	Peltier device	Heat sink	Heat sink	Water bath		Water bath	Fan
LED position relative to samples	Above	Below	Below	Above	Below	Below	Above
Sample volume^c^	200	200	0.027	200	1,000	1,000	200
Main purpose	Photosynthesis	Culture growth	Culture growth	Culture growth	Optogenetics	Culture growth	Photosynthesis

**Notes.**

a PAR irradiance (μmol photons m^−2^ s^−1^) operating intensity indicated if different from maximum.

b Peak wavelengths in the PAR region (nm).

c Working volume (ml).

d Wells too deep for fluorescence excitation, depending on the fluorometer design.

e Units not convertible to μmol photons m^−2^ s^−1^.

f Not LED-based but potentially adaptable for photosynthesis studies.

g Low number of fluorescence image pixels per sample.

h 2 LEDs per sample at a time, possibility to choose from 14 different colors in the PAR region.

The only exception is the ‘light plate apparatus’ (Gerhardt et al., 2016) that, using 24-well plates (which have wider wells and less shadowing effects) and relatively high levels of actinic light (>1,000 µmol photons m^−2^ s^−1^, adequate for realistic light stress conditions), makes it usable for multi-actinic and chlorophyll fluorescence imaging. However, this system is, like most others (Table 1), relatively expensive and, being based on custom-made electronics circuitry, requires significant engineering expertise to fabricate. Also, the effects of LEDs on sample temperature are uncertain (were not tested), and may be worsened by the fact that the LEDs are positioned below the samples. Other minor disadvantages include the smaller number of available colors per sample (which may be compensated by the large number of commercial available LED colors), the smaller number of samples (24), and the larger sample volumes required.

In addition to the aspects more specifically related to multi-actinic illumination and chlorophyll fluorescence detection, the illumination system described in the present study seems clearly advantageous to those listed, as it is based on low cost, open source electronics, 3D-printed parts, and off-the-shelf components. Both the microcontroller and the LED panel (the key parts of the system) can be replaced by similar models. Despite its simplicity, the Arduino Uno R3 was sufficient to control the illumination system, and this can be easily adapted or expanded to accommodate other microcontrollers. The LED panels used here are based on LED models widely used in electronics, with readily available documentation and other resources related to the manufacturing of components. As for the microcontroller, the LED panel may be easily replaced by other models—all that is needed is to adjust the dimensions of the well plate and of the LED spacer, which can be rapidly prototyped by modifying the 3D-printing files. Altogether, this device is inexpensive, low power consuming and easy to assemble by users without an engineering background, having the potential to rapidly increase research outputs through the parallel assessment of large numbers of samples.

### Further applications

In this study, the use of the illumination system was exemplified with samples of macroalgae. However, the system can readily be applied to study other types of samples, such as suspensions of microalgae (cultures of natural phytoplankton samples) or chloroplasts. It can be used with plant leaves, either in the form of leaf disks, as commonly done in plant physiology studies, often in combination with inhibitor treatments (Shen et al., 1996) or, if homogeneous and large enough, using single intact leaves (Serôdio et al., 2013).

Although the described system was devised for using chlorophyll fluorescence imaging to measure indices related to photophysiology and photosynthetic activity, it can also be used for a broader range of photosynthetic light responses, such as phototaxis of chloroplasts (Wada, 2013). Chloroplast movements have been shown to be of crucial photoprotective importance and can be monitored non-destructively using chlorophyll fluorescence (Park, Chow & Anderson, 1996; Kasahara et al., 2002) or through spectral reflectance (Berg et al., 2006), which can also detect similar processes that change the light absorption properties of samples. The presented illumination system can be applied to study such processes if combined with fluorometers that make use of bandpass filters (using a filter wheel) to determine reflectance indices related to chlorophyll content (e.g., Normalized Difference Vegetation Index) (Laviale, Frankenbach & Serôdio, 2016). While this study has emphasized the integration with imaging systems, because of the obvious advantages provided by the simultaneous measurement of multiple samples, the described system can also be used for non-image based approaches.

Another potential use of this system is the measurement of action spectra of photosynthetic responses. The availability of LEDs of different colors allows, although in a limited way, to study how photosynthetic processes respond to different light spectra, and thus enable a better characterization of organism-specific physiological responses (Glemser et al., 2016). In the particular context of the study of PSII photoinactivation, the use of LEDs of different colors can help address central questions regarding the primary mechanisms of PSII photoinactivation, namely the relative role of excess light energy vs inactivation of the Mn_4_CaO_5_ cluster of the PSII Oxygen Evolving Complex (the ‘photosynthetic pigment’ vs ‘Mn-cluster’ paradigms) (Zavafer, Chow & Cheah, 2015). Because the two mechanisms have very different spectral dependences, the comparison of *k*_*PI*_ (or its irradiance dependency, Φ_*PI*_) for blue, green and red can provide important information on the prevailing photoinactivation process. The application of actinic light of different colors may be especially relevant when comparing organisms with different pigment compositions, such as the algal groups used in this study.

The large number of LEDs per panel may also further enable the simultaneous study of the effects of light spectrum, intensity, and light dose. The fast response of LEDs also permit studies on the effects of intermittent and fluctuating light regimes, which are of interest for fundamental and applied research on photosynthesis and growth (Armbruster et al., 2017; Graham et al., 2017), and, more specifically, to characterize the differential response of PSII photoinactivation and repair processes under more realistic illumination conditions (Xu et al., 2016).

Finally, whilst the described system was designed for short-term light exposure experiments for characterizing photophysiological traits of photosynthetic organisms, it could be adapted to be used as part of a microphotobioreactor for studying microalgae culture growth. The spacing between individual LEDs on the LED panels used here match closely the spacing between wells of standard 96-well plates, making it easy to assemble a system similar to some listed in Table 1 (Chen et al., 2012; Gerhardt et al., 2016). Black 96-well plates with a transparent bottom could guarantee the independent illumination of adjacent wells and culture growth could be monitored in a standard plate reader. The LED panel could be used to illuminate the samples from above, as described, with the advantage of a more efficient heat dissipation, or from below, benefiting the exposure of the samples to higher light levels.

## Conclusions

This study presents a novel multi-actinic illumination system designed to promote the high-throughput characterization of photosynthetic responses to light stress. Combined with imaging chlorophyll fluorometry, it allows for the flexible control of intensity and duration of light exposure of multiple samples simultaneously, enabling the fast screening of the photosynthetic functional traits of a large number of samples exposed to ecophysiologically realistic light conditions. The system is inexpensive (total cost <150€) and simple to fabricate, and can be used to study a variety of photosynthetic samples, including microalgae suspensions, macroalgae or plant leaves, having the potential to be used for phenotyping and phenometric studies. The unique combination of low cost, easiness of fabrication, high light levels provided and usability with commercially-available imaging chlorophyll fluorometers makes this an interesting alternative to other available systems, not only for photosynthesis studies but potentially also as a basis for microphotobioreactors.

##  Supplemental Information

10.7717/peerj.5589/supp-1 Table S1 Spectral properties of the LEDs used in the study as a function of the PWM levels applied Contribution of violet (400–415 nm) < 0.04% in all cases (not shown). H1, wavelength of maximum intensity; B3, intensity (arbitrary counts) at wavelength of maximum intensity; FWHM, full width at half maximum.

10.7717/peerj.5589/supp-2 Supplemental Information 1 Arduino IDE code for experiment 1 Codes used for programming the Arduino microcontroller as used for experiment 1.

10.7717/peerj.5589/supp-3 Supplemental Information 2 Arduino IDE code for experiment 2 Codes used for programming the Arduino microcontroller as used for experiment 2.

10.7717/peerj.5589/supp-4 Supplemental Information 3 Arduino IDE code for experiment 3 Codes used for programming the Arduino microcontroller as used for experiment 3.

10.7717/peerj.5589/supp-5 File S1 STL file for part connecting fan to LED plate STL file for 3D-printing parts to fix the fan to the LED panel, and to fix the wires connected to the LED panel. M3 screws can be used to attach the two parts, enclosing and fixing the wires.

10.7717/peerj.5589/supp-6 File S2 STL file for LED spacer STL file for 3D-printing the LED spacer.

10.7717/peerj.5589/supp-7 File S3 STL file for well plate STL file for 3D-printing the custom-designed 64-well plate.

10.7717/peerj.5589/supp-8 File S4 STL file for PAR sensor holder STL file for 3D-printing the multi-position holder for the mini PAR sensor.

10.7717/peerj.5589/supp-9 Data S1 Raw data used for Figs. 2–6

## Additional Information and Declarations

### Data Availability

The following information was supplied regarding data availability: The raw data and arduino codes are provided in the Supplemental Files.
