# Supplementary material for: An LED-based multi-actinic illumination system for the high throughput study of photosynthetic light responses
Source: PeerJ. 2018 Sep 4;6:e5589. doi: 10.7717/peerj.5589 (PMC6128260; doi:10.7717/peerj.5589)
Supplement: Table S1 — Contribution of violet (400–415 nm) < 0.04% in all cases (not shown). H1, wavelength of maximum intensity; B3, intensity (arbitrary counts) at wavelength of maximum intensity; FWHM, full width at half maximum. [file peerj-06-5589-s001.docx]

Table S1. Spectral properties of the LEDs used in the study as a function of the PWM levels applied. Contribution of violet (400–415 nm) < 0.04% in all cases (not shown). H1: wavelength of maximum intensity; B3: intensity (arbitrary counts) at wavelength of maximum intensity; FWHM: full width at half maximum.

| PWM | PAR  µmol m^-2^ s^-1^ | Relative contribution to entire spectrum (%) | | | | Blue peak  400–500 nm | | | Green peak  500–605 nm | | | Red peak  605–700 nm | | |
| --- | --- | --- | --- | --- | --- | --- | --- | --- | --- | --- | --- | --- | --- | --- |
|  |  | Blue  400–510 nm | Green 510–550 nm | Yellow 550–605 nm | Red 605–700 nm | H1 | B3 | FWHM | H1 | B3 | FWHM | H1 | B3 | FWHM |
| 32 | 280 | 29.1 | 28.2 | 21.8 | 20.9 | 460 | 45.6 | 25 | 518 | 24.3 | 24 | 629 | 34.0 | 25 |
| 72 | 558 | 29.2 | 28.2 | 21.7 | 20.8 | 460 | 94.0 | 25 | 518 | 50.0 | 24 | 629 | 69.7 | 25 |
| 109 | 842 | 29.3 | 28.3 | 21.7 | 20.8 | 460 | 143.6 | 25 | 518 | 76.1 | 24 | 629 | 106.2 | 25 |
| 145 | 1116 | 29.4 | 28.3 | 21.6 | 20.7 | 460 | 192.0 | 25 | 518 | 101.8 | 24 | 629 | 140.5 | 25 |
| 182 | 1395 | 29.4 | 28.3 | 21.6 | 20.7 | 460 | 241.8 | 26 | 518 | 127.9 | 25 | 629 | 176.7 | 26 |
| 218 | 1665 | 29.4 | 28.3 | 21.6 | 20.7 | 460 | 288.0 | 26 | 518 | 152.2 | 25 | 630 | 210.4 | 26 |
| 255 | 1948 | 29.5 | 28.3 | 21.5 | 20.6 | 461 | 331.4 | 26 | 518 | 174.2 | 25 | 630 | 239.9 | 27 |
